# Optogenetic Control of Bacterial Cell‐Cell Adhesion Dynamics: Unraveling the Influence on Biofilm Architecture and Functionality

**DOI:** 10.1002/advs.202310079

**Published:** 2024-04-13

**Authors:** Juan José Quispe Haro, Fei Chen, Rachel Los, Shuqi Shi, Wenjun Sun, Yong Chen, Timon Idema, Seraphine V. Wegner

**Affiliations:** ^1^ Institute of Physiological Chemistry and Pathobiochemistry University of Münster Münster Germany; ^2^ Xiangya School of Pharmaceutical Sciences Central South University Changsha China; ^3^ Department of Bionanoscience Kavli Institute of Nanoscience Delft University of Technology Delft The Netherlands; ^4^ National Engineering Research Center for Biotechnology College of Biotechnology and Pharmaceutical Engineering Nanjing Tech University Nanjing China; ^5^ State Key Laboratory of Materials‐Oriented Chemical Engineering College of Biotechnology and Pharmaceutical Engineering Nanjing Tech University Nanjing China

**Keywords:** bacterial cell‐cell adhesion, biofilm, individual‐based simulations, optogenetics, photoswitchable proteins, quorum sensing

## Abstract

The transition of bacteria from an individualistic to a biofilm lifestyle profoundly alters their biology. During biofilm development, the bacterial cell‐cell adhesions are a major determinant of initial microcolonies, which serve as kernels for the subsequent microscopic and mesoscopic structure of the biofilm, and determine the resulting functionality. In this study, the significance of bacterial cell‐cell adhesion dynamics on bacterial aggregation and biofilm maturation is elucidated. Using photoswitchable adhesins between bacteria, modifying the dynamics of bacterial cell‐cell adhesions with periodic dark‐light cycles is systematic. Dynamic cell‐cell adhesions with liquid‐like behavior improve bacterial aggregation and produce more compact microcolonies than static adhesions with solid‐like behavior in both experiments and individual‐based simulations. Consequently, dynamic cell‐cell adhesions give rise to earlier quorum sensing activation, better intermixing of different bacterial populations, improved biofilm maturation, changes in the growth of cocultures, and higher yields in fermentation. The here presented approach of tuning bacterial cell‐cell adhesion dynamics opens the door for regulating the structure and function of biofilms and cocultures with potential biotechnological applications.

## Graphical abstract

In this study, the vital role of dynamic bacterial cell‐cell adhesion in biofilm formation is highlighted. Using photoswitchable adhesins and periodic dark‐light cycles, dynamic adhesions enhance bacterial aggregation, resulting in thicker biofilms, and improved functionality is shown. Manipulating adhesion dynamics with light offers a promising way forward for shaping biofilm structure and function with potential biotechnological applications.

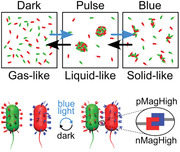

## 1 Introduction

Biofilms bring together different bacteria in communities that allow them to perform tasks, which their planktonic counterparts or a single species cannot accomplish.^[^ ^1^, ^2^ ^]^ Within the biofilm, bacteria share communal resources,^[^ ^3^ ^]^ divide tasks among the members and specialize,^[^ ^4^, ^5^, ^6^, ^7^ ^]^ communicate through quorum sensing^[^ ^8^, ^9^ ^]^ and are protected from environmental challenges, all increasing their chances of survival. Despite the well‐established relevance of biofilms in both diseases and ecology, as well as their growing relevance for biotechnological applications,^[^ ^7^, ^10^ ^]^ our ability to engineer them lags behind the advancements in engineering individual bacterial cells. Various biofilm properties, such as total biomass, spatial microstructure, compactness, the relative distribution of different members, and the dynamic evolution of these features over time, significantly impact biofilm performance.^[^ ^11^ ^]^ However, our current capabilities are primarily limited to analyzing these parameters rather than actively controlling them.

In biofilm maturation, bacteria initially form clusters known as microcolonies, which consist of tens to a few thousand cells.^[^ ^7^, ^12^, ^13^ ^]^ These microcolonies are not merely an intermediate stage in biofilm formation. They serve as the basic kernel for the subsequent microscopic and mesoscopic structure of the biofilm and alter the resulting functionality.^[^ ^14^, ^15^ ^]^ A major determinant of the microcolony and biofilm micro‐ and mesoscale architecture are the bacterial cell‐cell adhesions alongside other attractive and repulsive forces.^[^ ^11^, ^16^ ^]^ The strength of these cell‐cell adhesions and their dynamics (*t*_on_ and *t*_off_) can result in gas‐like, liquid‐like, and solid‐like multicellular organization analogous to different states of matter. Free‐floating bacteria in solution have negligible adhesions like molecules in gases. If the adhesions between the cells get stronger but are still dynamic, bacteria are still mobile within the aggregates like molecules in a liquid. On the other hand, if the interactions between cells are strong and not dynamic, the cells will stay in place like molecules within a solid matrix. This has consequences for bacterial behavior.^[^ ^16^ ^]^ As shown in *V. cholerae*, lowering the cell‐cell adhesions results in larger cell‐cell distances in biofilms.^[^ ^11^ ^]^ Moreover, in these rod‐shaped bacteria, the cell‐cell adhesions also influence nematic order reflected in the roughness of the forming colony.^[^ ^17^, ^18^ ^]^ In other cases bacterial microcolonies behave like liquids, where they have short‐range but no long‐range order, form spherical colonies to minimize surface tension, and fuse with each other when they come in contact.^[^ ^19^, ^20^ ^]^ The liquid‐like dynamics are also important for cell sorting in colonies based on the differential strength of adhesions, as the sorting requires some level of cell mobility.^[^ ^11^, ^13^, ^21^ ^]^ Moreover, whether bacteria exhibit liquid or solid‐like properties impacts their ability to colonize capillaries^[^ ^22^ ^]^ and their antibiotic susceptibility.^[^ ^23^ ^]^

Regulating the dynamics of bacterial cell‐cell adhesions and the transitions of bacteria between the gas, liquid, and solid‐like states could provide a viable approach to addressing the challenge of engineering biofilm development, yet this avenue remains largely unexplored. The sensitivity of cell‐cell interactions to the dynamics and equilibration of cell‐cell adhesions makes them challenging to control experimentally. Current studies predominantly rely on the manipulation of gene expression^[^ ^24^ ^]^ and genetic modification of adhesin properties.^[^ ^11^, ^19^ ^]^ Conversely, various initiatives aim to introduce synthetic adhesins that are either genetically coded (e.g. nanobody/antigen,^[^ ^24^ ^]^ SpyTag/SpyCatcher^[^ ^25^ ^]^) or chemically introduced (e.g. click chemistry^[^ ^26^ ^]^). Notably, optogenetic tools enabling light‐inducible expression of adhesins have enabled the production of micropatterned biofilms.^[^ ^27^, ^28^, ^29^, ^30^, ^31^ ^]^ However, all these adhesions lack reversibility, rely on strong and permanent interactions, and are not dynamic. In contrast, cell‐cell adhesions between bacteria are dynamic in many instances. For example, many bacterial species use type 4 pili (T4P) to generate attractive forces between cells, with the pili known to govern liquid‐like behavior in microcolonies.^[^ ^16^ ^]^ Here, T4P binds to pili on neighboring bacteria and continuously retracts, resulting in the observed liquid‐like behavior.

In this study, we elucidate how the strength and dynamics of bacterial cell‐cell adhesions influence bacterial aggregation, as well as the architecture and function of the resulting biofilms. Using photoswitchable adhesins, we were able to systematically modify the dynamics of bacterial cell‐cell adhesions and switch between gas‐like, liquid‐like, and solid‐like multicellular aggregates. Our findings reveal that similar to distinct states of matter, bacterial aggregates, and biofilms possess unique mechanical and biological properties that depend on the cell‐cell adhesion dynamics. Consequently, we propose that precise regulation of adhesion dynamics within microcolonies holds significant potential in guiding future strategies for biofilm engineering, as we have exemplified in auxotrophic cocultures and a biofilm reactor.

## 2 Results and Discussion

### 2.1 Pulsed Light Illumination to Control Bacterial Adhesion Dynamics

**Figure 1:**
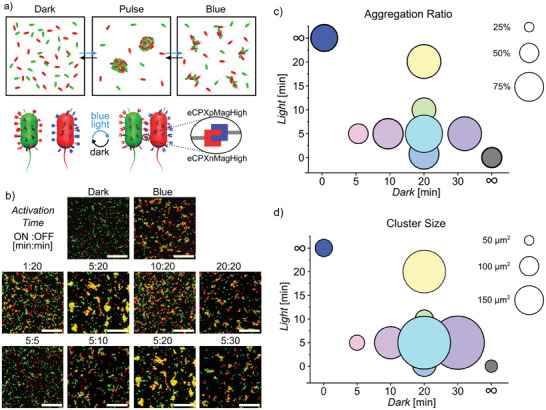
Pulsed illumination alters aggregation ratio and cluster size. a) Schematic representation of bacteria expressing nMagHigh‐eCPX (labeled green) and pMagHigh‐eCPX (red) showing different aggregation levels under different pulse illumination settings. b) Confocal microscopy images of bacteria incubated in the dark, under blue light or different pulsed illumination settings (ON:OFF time in minutes) for a total duration of 2 h, show different amounts of aggregation. Images at 5:20 are shown twice to make trends visible but come from the same experiment. Scale bars are 50 µm. Differences in c) aggregation ratio and d) cluster size for different illumination settings. In terms of both aggregation ratio and cluster size, some pulsed light settings outperformed constant blue light illumination. All experiments were performed in 3 biological replicates with 3 technical replicates each.

In this study, we employed blue light switchable bacterial cell‐cell adhesions to manipulate adhesion dynamics and consequently control bacterial aggregation into multicellular structures. Specifically, the bacteria could undergo transitions from a gas‐like state characterized by weak attractive interactions between cells to a liquid‐like state where dynamic cell‐cell adhesions enable cell mobility within aggregates, and finally to a solid‐like state with strong and static cell‐cell adhesions with rigid and unchanging internal configurations (**Figure** **1**a). The modulation of cell‐cell adhesion strength and dynamics was achieved through the expression of photoswitchable proteins, namely nMagHigh or pMagHigh, as adhesins on the outer surface of *E. coli*.^[^ ^32^, ^33^ ^]^ nMagHigh and pMagHigh proteins have been derived from the light‐dependent homodimerize VVD from *Neurospora crassa* and have been engineered to expose complementary proteins interphases with negatively (I52D and M55G for nMagHigh) and positively (I52R and M55R for pMagHigh) charged amino acids, respectively, under blue light.^[^ ^34^ ^]^ This happens due to the blue light‐triggered reaction between a key cysteine (C71) reacts with the flavin dinucleotide chromophore, resulting in global conformational changes. The complementary electrostatic interaction between nMagHigh and pMagHigh promotes selective heterodimerization and prevents undesired homodimerization. In our case, these adhesins were displayed on the outer bacterial membrane as fusion proteins with the circularly permutated outer membrane protein OmpX (eCPX). In the dark, nMagHigh and pMagHigh bacteria did not adhere to each other, resembling a gas‐like state. On blue light illumination, these two types of bacteria permanently adhered to each other, reminiscent of a solid‐like state. Notably, these optogenetic adhesins are reversible in the dark and can be repeatedly switched on and off using pulsed light illumination. The photoactivation of the adhesins occurs within seconds,^[^ ^33^, ^34^ ^]^ and the rate‐limiting step for aggregation is bacteria coming in close proximity in suspension. In our previous work, we observed that the bacterial aggregation plateaus within about 2 h (*t*_1/2_ ≈ 30 min) at densities also used in this study. At the molecular level the reversion rate of the nMagHigh‐pMagHigh interaction in the dark is significantly slower (*t*_1/2_ = 4.7 h),^[^ ^34^ ^]^ also resulting in slow reversion rates of the bacterial aggregates in the dark within about 1 h.^[^ ^32^, ^33^ ^]^

To investigate the influence of cell‐cell adhesion dynamics on bacterial aggregation, we mixed an equal number of *E. coli* MG1655 bacteria expressing nMagHigh (labeled with green fluorescent protein GFP, shown in green) and pMagHigh (labeled with mCherry, shown in red) in PBS and exposed them to various blue light illumination patterns (450 nm blue LED, 270 µW cm^−2^) for a total duration of 2 h. During this time, there was minimal bacterial growth, excluding the effects of crowing. These mixtures of bacteria aggregated in different manners in terms of aggregation ratio (area occupied by clusters with an area >35 µm^2^ divided by the total area occupied by all bacteria), average cluster size (objects with an area >35 µm^2^), and total number of clusters depending on the illumination as observed with confocal microscopy (Figure 1b; Figure S1d, Supporting Information). Samples kept in the dark serve as a negative control, showing weak interactions and low levels of background aggregation (gas‐like) (Few clusters with >35 µm^2^ were detected in the automated image analysis due to local crowding, i.e., cells statistically being in close proximity due to their overall density). Samples exposed to continuous blue light illumination served as a positive control with strong and static adhesions (solid‐like).

Initially, we kept the dark period constant at 20 min, altering the illumination period from 1 to 20 min. With a brief 1‐min illumination time, we still observed comparable aggregation to constant blue light illumination. Even more strikingly, there was a significant rise in aggregation ratio and average cluster size at 5 and 20 min illumination periods compared to continuous blue light illumination, despite overall lower light exposure (Figure 1c; Figure S1a, Supporting Information). Intriguingly, 10‐min photoactivation periods resulted in a reduction in aggregation ratio and average cluster size. In a second set of experiments, we fixed the light period to 5 min, varying the dark period from 5 to 30 min. In this scenario, the aggregation ratio increased compared to continuous blue light illumination whenever the dark period exceeded 10 min. Concurrently, the average cluster size also increased as the dark period was extended from 5 to 20 min (Figure 1d; Figure S1c, Supporting Information). In these experiments, a light period of 5 min together with a dark period of 20 min was observed to be optimal for reaching the highest aggregation ratio and the largest cluster size.

### 2.2 Liquid‐Like Behavior in Bacterial Aggregates

**Figure 2:**
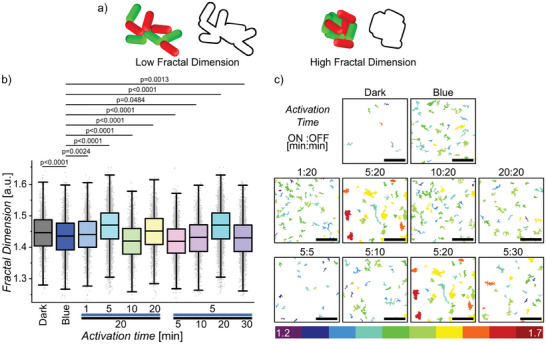
Cluster morphology varies under different illumination settings. a) The fractal dimension quantifies the compactness of the clusters, branched and irregular clusters have a low fractal dimension while more regular clusters have a higher score. b) Distribution of bacterial clusters’ fractal dimension from different pulse illumination settings (ANOVA with multiple comparisons, *p* < 0.0001). c) Visual categorization of bacterial clusters according to fractal dimension, the key below shows the color code of clusters’ respective fractal dimensions from 1.2 to 1.7 with 0.05 increments. ON: OFF time in minutes (total time 2h, scale bars 50 µm, all experiments were performed in 3 biological replicates with 3 technical replicates each.). Data and images in (b) and (c) at 5:20 are shown twice to make trends visible but come from the same experiment.

In addition to the clustering efficiency of the bacteria, the aggregate morphology also changes significantly depending on the illumination protocol. The clusters exhibit a wide range of structures, varying from branched structures to more compact and spherical ones. To assess the morphology, we analyzed the fractal dimensions of the clusters using the FracLac plugin in ImageJ (**Figure** **2**a). In this analysis, more compact objects have a higher fractal dimension than ones that are less compact with an irregular shape. The median fractal dimension was highest for aggregates under 5 min on and 20 min off light illumination (5:20), measuring 1.477. In comparison, under continuous blue light illumination, the median fractal dimension was only 1.443 (Figure 2b,c). The initial stages of microcolony formation exhibited striking similarities to the self‐assembly of non‐living colloidal particles with attractive interactions. This resemblance is particularly expected since the nMagHigh and pMagHigh expressing bacteria in PBS showed low motility. In the self‐assembly of colloids, the resulting architectures depend on the dynamics of the interactions. When the interactions are static, diffusion‐limited cluster aggregation (DLCA) leads to loosely packed and branched assemblies under kinetic control. Similarly, in our experiments under constant blue light illumination, the rate‐limiting step involved different bacteria finding each other, resulting in branched aggregates, i.e., lower fractal dimension. In contrast, dynamic interactions enable reaction‐limited cluster aggregation (RLCA) where colloids optimize their position to maximize the interactions with neighbors, leading to compact and spherical structures under thermodynamic control. In our experiments, pulsed light illumination introduced dynamics into the bacterial cell‐cell adhesions and increased the fractal dimension, thereby shifting the bacterial assemblies from DLCA under constant light illumination towards RLCA (Figure S2, Supporting Information). As a benchmark, ideal conditions for 2D aggregation of spherical particles yield fractal dimensions of 1.46 and 1.55 for DLCA and RLCA, respectively.^[^ ^35^, ^36^ ^]^

Our comprehensive analysis of aggregation and fractal dimension data leads us to propose a hypothesis regarding the impact of bacterial cell‐cell adhesion dynamics. We predict that during the dark periods, the photoswitchable adhesions responsible for holding the clusters together gradually reverse, but not completely if the dark period is lower than the reversion time. Consequently, bacteria residing at the periphery of the clusters that are only weakly attached could escape or reposition themselves, enabling stronger interactions once illumination resumed. These enhanced attachment and detachment dynamics among bacteria—akin to a liquid‐like state—result in larger and rounder aggregates. Under constant illumination, this dynamic behavior is absent (i.e. solid‐like state), and adhesions occur rapidly among neighboring cells. These cells thus form only small clusters with permanent interactions, absorbing all free‐floating bacteria that could contribute to larger clusters. Insufficient reversion, as seen with a short dark period (e.g., 5 min), results in an insignificant improvement in bacterial aggregation, as the bacteria lack the motility to reposition. On the other hand, when the dark period extends to 30 min, causing extensive reversion, both the aggregation ratio and the average cluster size decrease, as the cells return to a gas‐like state.

### 2.3 Individual‐Based Simulations of Bacterial Aggregation

**Figure 3:**
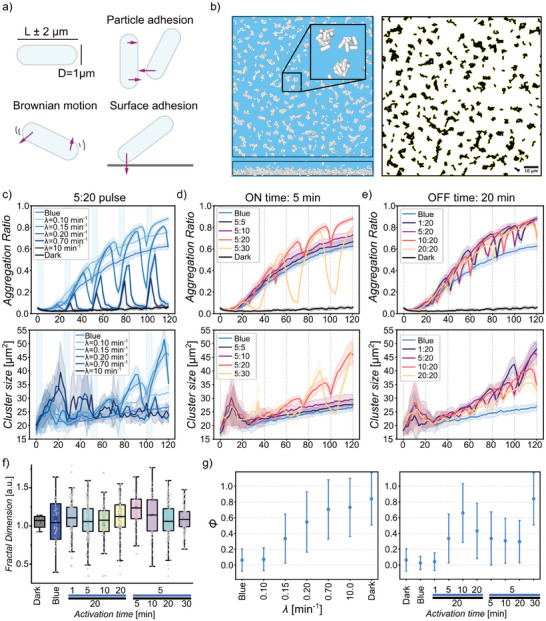
Individual‐based simulations. a) The bacteria are modeled as spherocylindrical particles. Each timestep, Brownian force, and adhesive forces to the surface and to other particles are exerted for each particle. b) Still of a simulation of 2000 particles after 1 h of constant light (top view and side view) and the resulting 2D image and segmentation used for assessing the aggregation ratio and cluster size. c) Aggregation dynamics and cluster sizes under a pulse illumination of 5:20, with varying decay rates of the adhesive force. Averages and standard deviation of 10 runs per condition are shown. d,e) Aggregation dynamics and cluster sizes under different pulse illumination settings. Averages and standard deviation of ten runs per condition are shown (f) Distribution of the fractal dimension of particle clusters for different pulse illumination settings. (ANOVA with multiple comparisons, *p* = 0.0005.) (g) Neighbor rearrangement of the particles under 5:20 pulse illumination for different values of the decay rate, *λ*, and for different pulse illumination settings with *λ* = 0.15 min^−1^.

To further investigate the dynamics of bacterial clustering, we used an individual‐based model of spherocylindrical particles to simulate the experiments. Besides Brownian forces, each particle is influenced by light‐sensitive interactions with other particles and interactions with a hard surface (**Figure** **3**a). After initialization, the system evolved over time, and particle locations were tracked and analyzed like in the experiments (Figure 3b). We determined the magnitude of the Brownian forces by estimating the diffusion coefficient for particles of similar size, shape, and environment as the bacteria in the experiments and comparing the expected mean‐squared‐displacement (MSD) to the MSD of simulated particles with no adhesive forces (Figure S3, Supporting Information). With the correct Brownian forces in place, we estimated the magnitudes (*P* and *S*) and ranges (α and *β*) for the adhesive forces between the particles and the surface, respectively, by comparing aggregation curves from the simulations to those from experiments with constant light illumination^[^ ^33^ ^]^ (Figure S4, Supporting Information).

In the model, the part of the force that is affected by the blue light is the magnitude of the particle‐particle interaction, *P*, which is constant under blue light (*P_on_*). However, the adhesive force is not immediately set to zero when the light is turned off. Instead, after each period of light exposure, it follows an exponential decay with the decaying adhesive force of the form *P = P_on_ · e^−λ·t^* where *P_on_* is the force magnitude under blue light, *λ* is the decay rate and *t* is the time since the light was turned off in minutes. We found the value for *λ* by fitting it to the 5:20 pulse illumination experiments, where the aggregation ratio was the highest (Figure 3c). For high decay rates (*λ* = 10 min^−1^), we observed a complete reversal of the clustering in the periods of darkness, which led to low aggregation ratios like in the dark and no increase in cluster size over multiple cycles of illumination. For low decay rates (*λ* = 0.1 min^−1^), there was a persistent rise in aggregation ratio and cluster size over multiple cycles of illumination, which is slightly higher than what we find for constant blue light. The highest aggregation ratio was achieved when the clusters started to disassemble just before the light was turned back on (*λ* = 0.15 min^−1^).

When using this value for other illumination conditions we could recreate most of the findings of the experiments, where we clearly observed higher aggregation ratios and bigger cluster sizes for all the pulsed illumination conditions compared to constant blue light (Figure 3d‐e; Video S1–S3, Supporting Information). For conditions with an on‐time of 5 min, we see that for short off‐times up to 10 min, the aggregation and cluster size only slightly increased compared to constant blue light illumination, and the curves remained smooth during the pulsed light illumination (Figure 3d). However, for an off‐time of 20 min there was significant disassembly during dark periods, resulting in a drastic growth of the clusters when the light was turned back on. When the off‐time was increased even more, we saw an an extensive reversal of the clustering, which explains the lower aggregation ratios and smaller clusters found in the experiments under these conditions.

The conditions with off‐times of 20 min consistently showed higher aggregation levels and bigger clusters compared to constant blue light illuminations, just like in the experiments (Figure 3e). In contrast, the on‐time did not seem to globally affect the aggregation ratio as all curves behaved similarly, but only the cluster size was affected, where longer on‐times led to smaller clusters. We also find that for these conditions, the time of measurement can significantly affect the result, which could explain why the 10:20 and 20:20 conditions gave such varying results in the experiments, as both illumination conditions are right at the end of a period of darkness at the time of measurement resulting in a seemingly low aggregation ratio and smaller clusters. Analogous to our experimental results, the fractal dimension of the clusters formed under pulse illumination in the simulations was also higher than that of clusters formed under constant blue light and in the dark (Figure 3f). All in all, these results demonstrate that in our experimental system under pulse illumination, the off‐time of 20 min was the critical parameter, which provides just enough time for the clusters to start to disassemble before the light is turned back on. This partial disassembly in turn leads to the particles rearranging significantly and the clusters coming together again in bigger structures leading to higher aggregation ratios and cluster sizes.

To explore how this rearrangement on the particle scale relates to the overall aggregation dynamics, we looked at the neighbor rearrangement of the particles during a 5:20 pulse cycle. For each particle, we tracked which particles were in its close vicinity throughout the simulation. We then compared the neighbors at the beginning and end of one ON: OFF cycle and calculated the rearrangement *φ* as a value between 0 and 1, in the following way,

(1) $$\varphi \;=\;1\;-\frac{1}{N}\sum_{i}\frac{\left|\;n_{i,\;\mathrm{beginning}}=\;n_{i,\;\mathrm{end}}\right|}{\left|n_{i,\;\mathrm{end}}\right|}$$

where *N* is the number of particles and *n_i,t_* is the vector of neighbors of particle *i* at time *t*.

For the 5:20 pulsing scenario, we looked at the rearrangement for different values of the decay rate. For *λ* = 0.15 min^−1^, where we observed the highest aggregation ratio and biggest cluster sizes, we found an average rearrangement of ≈35% of neighbors per 25 min cycle (Figure 3g). In contrast, rearrangement between cycles was almost complete (≈85%) for the higher values of *λ* and in the dark but rearrangement remained below 20% for lower values of *λ* and under constant blue light illumination. Similarly, when comparing different illumination conditions, we saw that also here, the highest aggregation ratios correspond to a rearrangement of ≈35% of neighbors in 25 min. In summary, the highest aggregation is achieved with intermediate dynamics in the neighbor rearrangement, resulting in liquid‐like behavior. Here, the timescale of the disassembly process is similar to the off‐time in the pulse illumination. Because the disassembly timescale is intrinsic to the system, it is possible to find a pulse condition where the aggregation is optimal by tuning off‐time.

### 2.4 Bacterial Cluster Intermixing

**Figure 4:**
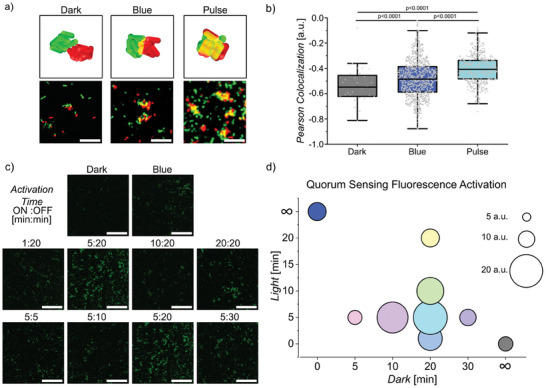
Bacterial mixing and quorum sensing. a) Preformed aggregates of *E. coli* expressing nMagHigh and pMagHigh labeled with GFP (shown in green) or mCherry (shown in red) were mixed and allowed to further interact under different illumination settings. Scale bars are 30 µm. b) Clusters formed under 5:20 pulse illumination, showed more intermixing of GFP and mCherry expressing bacteria than under blue light or in the dark as confirmed with a higher PCC, p‐values from t‐tests. c) Confocal microscopy images of *E. coli* with a quorum sensing GFP reporter plasmid and expressing nMagHigh or pMagHigh at their surface under different illumination settings. Scale bars are 50 µm. d) Average GFP fluorescence of bacteria in (c). Images in (c) at 5:20 are shown twice to make trends visible but come from the same experiment. All experiments were performed in 3 biological replicates with 3 technical replicates each.

Besides the rounding up of the aggregates with increasing bacterial adhesion dynamics, liquid‐like behavior is also characterized by the intermixing of similar liquids. To demonstrate that this aspect of liquid‐like behavior holds true in the bacterial aggregates, we mixed preformed aggregates of bacterial clusters under different illumination conditions. In particular, we performed two populations of compact bacterial clusters from nMagHigh‐ and pMagHigh‐bacteria either labeled with GFP (shown in green) or mCherry (shown in red) for 2 h under pulsed illumination (5:20 ON:OFF, referred to as pulsed light hereafter, if not specified otherwise). Subsequently, we mixed these clusters in equal numbers and kept them for another 2 h in the dark, under continuous or pulsed light illumination, before analyzing the intermixing of the GFP and mCherry labeled populations using confocal microscopy (**Figure** **4**a). In the dark, the red and green bacteria were predominantly dispersed due to the reversion of the adhesions and only a few clusters of a single color remained. In contrast, in samples kept under blue light, we observed clusters of green and red labeled bacteria with adjacent green and red domains, suggestive of clusters aggregating together. For samples kept under pulsed light, we also observed clusters, which consisted of blended red and green‐labeled bacteria, showing substantial intermixing of the two populations. We supported these qualitative observations by calculating the Pearson Correlation Coefficient (PCC)^[^ ^37^ ^]^ for the green and red channels (Figure 4b). The PCC was highest for samples under pulsed illumination, indicating a high colocalization of the red and green channels. The PCC was lowest for samples kept in the dark, indicative of only background‐level interactions between the green and the red labeled bacteria. Overall, both the rounder and more compact bacterial aggregates (Figure 2) and the higher degree of intermixing with pulsed light illumination support the higher fluidity of these bacterial aggregates compared to ones formed under constant illumination.

### 2.5 Consequences of Liquid‐Like Behavior for Quorum Sensing

Bacteria possess the ability to sense their local population density through quorum sensing.^[^ ^38^ ^]^ The activation of quorum sensing is highly contingent on the spatial distribution of bacteria, with compact aggregates locally reaching the threshold concentration of the autoinducer earlier than loose structures. To investigate the impact of bacterial aggregation on quorum sensing, we co‐transformed bacteria with the photoswitchable adhesins (nMagHigh or pMagHigh, 1:1 ratio) and a quorum sensing reporter plasmid, triggering the production of GFP when quorum sensing is activated. After incubating these co‐cultures (OD_600 _= 0.12) for 2 h, we observed comparatively lower quorum sensing reporter activity in samples kept in the dark compared to those under continuous blue light illumination (Figure 4c; Figure S1b, Supporting Information). Remarkably, samples subjected to pulsed illumination (5:20 or 5:30) exhibited a two‐fold increase in reporter signal compared to samples exposed to constant blue light. Conversely, samples kept under pulsed illumination with shorter periods of darkness (5 min off) or with shorter or longer durations of light showed similar levels of quorum sensing reporter as the constant blue light sample (Figure 4d). Notably, the more intense reporter signals were localized to larger bacterial aggregates. Overall, the activation of quorum sensing correlated with both cluster size and fractal dimension.

### 2.6 Biofilm Formations is Influenced by Bacterial Cell‐Cell Adhesion Dynamics

Bacterial aggregation constitutes an early and pivotal stage in biofilm formation, with these microcolonies serving as structural units that prime the subsequent maturation of the biofilm. To investigate the impact of bacterial cell‐cell adhesion dynamics on biofilm mesostructure and maturation, we produced biofilms using 1:1 cocultures of *E. coli* MG1655 expressing adhesins nMagHigh (co‐expressing cyan fluorescent protein, eCFP, depicted in green) and pMagHigh (co‐expressing yellow fluorescent protein, eYFP, depicted in red) for 48 h at 37 °C under various illumination conditions. (eCFP and eYFP, being point mutants of each other, are expected to impose an equal metabolic burden.)

**Figure 5:**
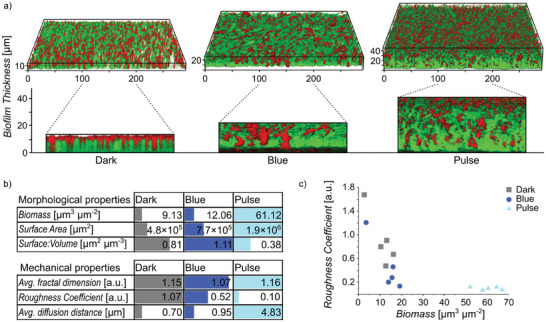
Pulse illumination enhances biofilm formation and properties. a) 3D and side view confocal microscopy images of biofilms formed with cocultures of nMagHigh‐ and pMagHigh‐ bacteria (shown in green and red, respectively) after 48 h under dark, blue, and pulsed illumination. b) COMSTAT analysis of biofilms. Both mechanical and morphological properties of biofilms were improved under pulsed illumination, resulting in a more massive and compact biofilm. c) Biofilms grown in pulse illumination have a higher biomass and lower surface roughness compared to those grown in the dark or under continuous blue light. (*n* = 5, each independent experiment is shown as one point).

We found that biofilms grown under pulsed illumination exhibited the highest overall biomass and denser structures compared to those formed under continuous blue light illumination or in the dark, as evidenced by confocal microscopy (**Figure** **5**a). In addition, biofilms formed under continuous blue light illumination were thicker than those formed in the dark. Specifically, the average thickness was 16, 31, and 42 µm for biofilms formed in the dark, under continuous blue light and pulsed blue light, respectively. These observations were further supported by crystal violet staining of biofilms in microplates, demonstrating higher total biomass under pulsed light illumination (Figure S5, Supporting Information).

A detailed analysis of the biofilms, encompassing mesostructure, surface properties, and bacterial distribution, was conducted using the automated image analysis tool for biofilms, Comstat 2, revealing pronounced morphological differences (Figure 5b; Figure S6, Supporting Information). Biofilms grown under pulsed light exhibited significantly higher biomass, with a 6.7‐ and 5.1‐fold increase compared to biofilms grown in the dark and under constant blue light illumination, respectively. Furthermore, these biofilms formed under pulsed light displayed smoother surfaces and higher packing density compared to biofilms formed in the dark and under continuous blue light, as indicated by a lower surface‐to‐volume ratio, a higher average fractal dimension, and a lower roughness coefficient (Figure 5c). These findings align with our earlier observations of denser bacterial clusters forming under pulsed light. Additionally, biofilms formed under pulsed light exhibited 7‐ and 5‐fold higher average diffusion distances compared to those formed in the dark and under continuous blue light, indicating enhanced connectivity between microdomains.

Drawing an analogy to states of matter, biofilms formed under pulsed illumination—where bacteria exhibit liquid‐like behavior—demonstrated enhanced intermixing. Like in bacterial aggregation, liquid‐like behavior played a role in bacteria optimizing their positions to maximize contact with neighbors by filling gaps within the biofilm. In contrast, biofilms formed under continuous blue light exhibit solid‐like characteristics, resulting in rougher biofilms. It is noteworthy that strong and permanent bacterial cell‐cell adhesions do not lead to more mature biofilms; rather, the more dynamic and transient adhesions result in enhanced biofilm maturation.

### 2.7 Photoregulation of Bacterial Consortia

In biofilms, diverse bacterial species often come together with various social interactions^[^ ^39^ ^]^ and such co‐cultures bear significant technological importance. Yet, effectively controlling, maintaining, and optimizing these biofilm cocultures is extremely challenging because their success hinges upon numerous factors, including the interplay between different members, the costs and benefits for each participant, the spatial distribution of members, and the availability of nutrients in the medium over time. Consequently, community members within the biofilm adapt their positions based on their needs, relationships (mutualistic, parasitic, commensal, etc.), and environmental factors.^[^ ^40^ ^]^ We propose that manipulating the illumination conditions can alter the architecture and success of different co‐culture biofilms, providing a means of optimization.

**Figure 6:**
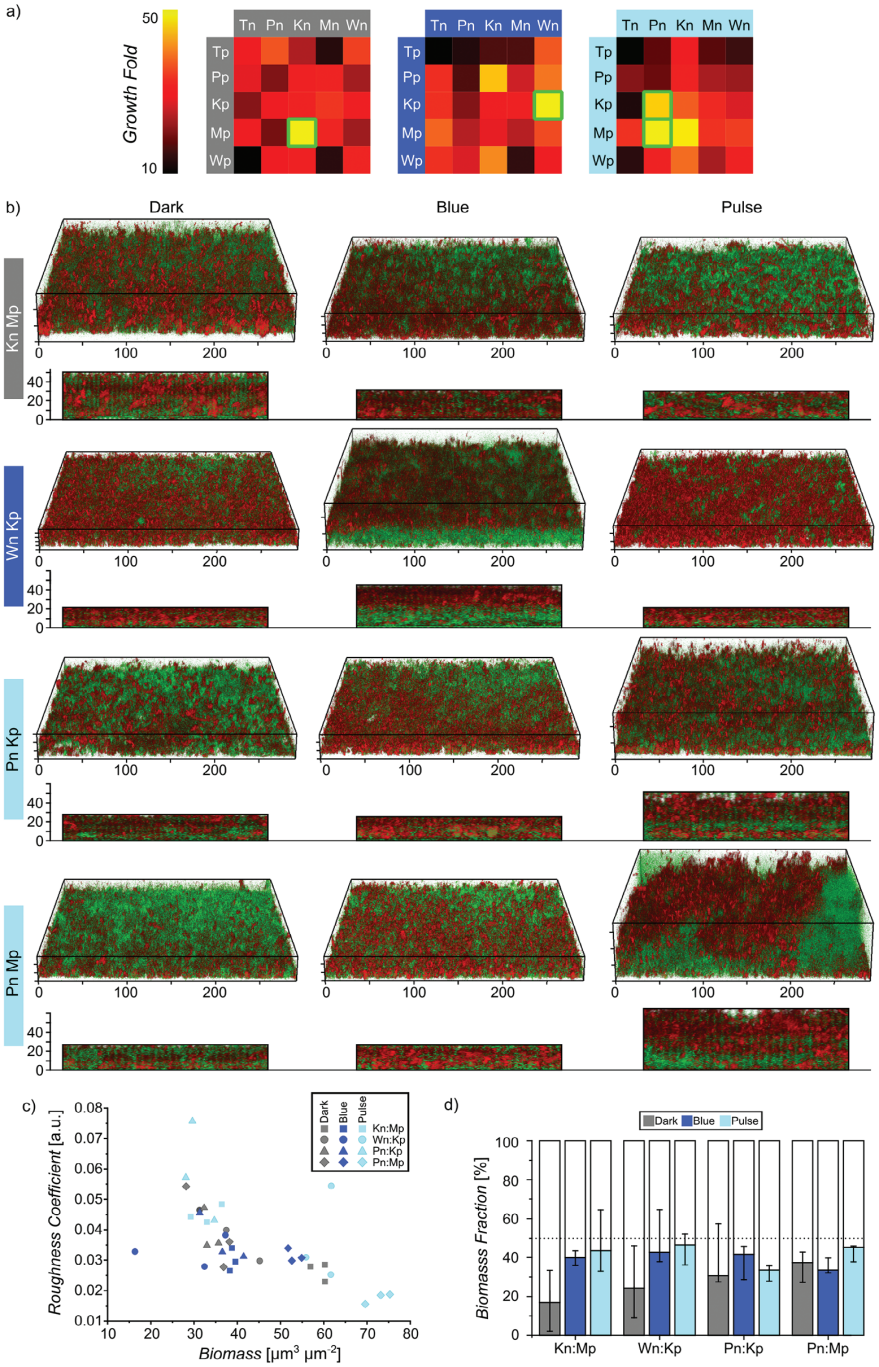
Pulsed illumination affects metabolic cooperation pathways in cross‐feeding cultures. a) Growth of cocultured auxotrophic strains displaying nMagHigh or pMagHigh after 48 h under different illumination settings in suspension culture. Cocultures thrive in different illumination conditions outlined in green. The experiments were conducted in three biological replicates. b) 3D and side view confocal microscopy images of selected coculture pairs under different illumination settings. nMagHigh and pMagHigh expressing bacteria are shown in green and red, respectively. Scale bars in µm. c) Surface roughness versus biomass for different cocultures under various illumination conditions. (*n* = 3, each point represents one sample) d) Biomass fraction of nMagHigh (shaded) and pMagHigh (white) for different cocultures that all started at equal numbers. Error bars represent the SD of three biological replicates.

To investigate this, we introduced the nMagHigh or pMagHigh adhesins onto auxotrophic strains lacking the ability to synthesize threonine (T), proline (P), lysine (K), methionine (M), or tryptophan (W), relying instead on another member of the community to produce these amino acids for them.^[^ ^41^, ^42^ ^]^ The nomenclature of the ten new strains was derived from the amino acid they were unable to produce (T, P, K, M, or W) and the type of adhesin they displayed (n or p). For example, Kn denoted a lysine auxotroph expressing the nMagHigh adhesin. In addition, nMagHigh and pMagHigh expressing strains were labeled with eCFP and eYFP, respectively. Subsequently, we co‐cultured the various autotrophic pairs possessing complementary photoswitchable adhesins in minimal M9 medium in the dark, under constant or pulsed blue light illumination. After 48 h of incubation at 37 °C in suspension culture, some pairs demonstrated successful cross‐feeding and thrived, showing synergistic growth as monitored by the OD_600_ (optical density at 600 nm) (**Figure** **6**a). It was intriguing to observe that different co‐cultures grew better under specific illumination conditions. The Kn‐Mp pair displayed significant growth exclusively in the dark, while the Wn‐Kp pair exhibited the highest growth under constant blue light illumination. Contrarily, pulsed light illumination was most favorable for the growth of the three pairs Pn‐Kp, Pn‐Mp, and Kn‐Mp. These initial findings underscore the fact that there is not one procedure that fits all co‐cultures and factors beyond bacterial cell‐cell adhesion dynamics may be decisive. Nevertheless, in certain cases, cell‐cell adhesions prove to be crucial, enabling the adjustment of proximity between members and balancing the cost of producing a common good with the benefit derived from receiving metabolites from the other member. Yet, these seem to be extremely sensitive to the precise context, and differences in the metabolic adaptations of the autotrophs seem to of significant. For instance, even switching the places of nMagHigh and pMagHigh, which only differ in two amino acids (D52R and G55R), between the two auxotrophic strains pair altered the outcome.

To examine the differences in biofilm architecture for these auxotrophic pairs, we formed biofilms using pairs that showed significant differences in growth depending on the illumination (Figure 6b). Different co‐cultures formed the thickest biofilms and showed different mesostructures under different illumination conditions, aligning with results from the growth analysis in suspension culture. The Kn‐Mp pair formed the thickest biofilms in the dark, almost twice as thick as under continuous or pulsed light. The Wn‐Kp pair grew most successfully under continuous blue light, forming a layered biofilm (Wn at the bottom shown in green, Kp at the top shown in red). Finally, the Pn‐Kp and Pn‐Mp pairs grew the thickest biofilms under pulsed light illumination, with the Pn‐Kp strains mixing within the biofilm and the Pn‐Mp pair forming a layered biofilm. These differences in biofilm architecture were also visible in the COMSTAD analysis (Figure S7, Supporting Information), where the thicker biofilms came along with a lower roughness coefficient and higher biomass (Figure 6c). It is also notable that we were able to regulate the ratio of each constituent strain of bacteria with different illumination settings (Figure 6d). While we initially mixed the nMagHigh and pMagHigh expressing strains in equal numbers, the biomass fraction of each member changed with different illumination settings. In some cases, the ratio changed significantly to form the most successful biofilm, as seen in the Kn‐Mp culture in the dark where the former outnumbers its neighbor in an 8:2 ratio. In contrast, in the biofilm of Pn‐Mp, which grew the best under pulsed light illumination, the ratio remained the same as the initial seeding condition. In the case of blue light and pulse illumination, the higher growth may be linked to a more efficient metabolic exchange between the two strains due to stronger cell‐cell adhesions. Yet, in this optimization process, bacterial motility and hence the fluidity within the biofilms at certain stages appear to be important factors. Microbial industrial fermentations usually rely on cocultures of different bacteria strains to carry out the energetically costly synthesis of metabolites.^[^ ^43^ ^]^ However, minor differences in factors such as growth rate, inoculation density, or preculture age of bacteria used in such processes, lead to the faster member of a coculture outcompeting the others and establishing a monoculture.^[^ ^44^ ^]^ The described photoswitchable adhesions and different illumination protocols may be used to counteract these challenges.

### 2.8 Pulsed Light Promotes the Production of *L*‐Threonine in Immobilized Fermentation

Biofilm bioreactors are of great industrial importance for the production of high‐value metabolites. A previous study has already demonstrated that continuous photoactivation of nMagHigh‐pMagHigh surface adhesins enhances the production yield of *L*‐threonine in immobilized continuous fermentation using *E. coli* W1688.^[^ ^45^ ^]^ Here, we hypothesized that pulsed illumination could further improve biofilm formation, fermentation, and bioproduction.

**Figure 7:**
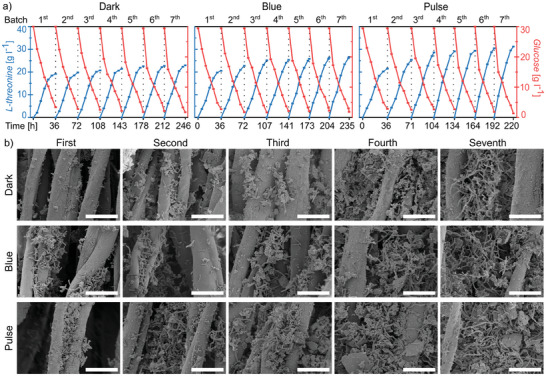
Pulse illumination increases biomass and positively affects *L*‐threonine production and glucose consumption in immobilized continuous fermentation. a) Along seven fermentation batches, 5:20 pulsed illumination increases the overall yield of *L*‐threonine and reduces the batch duration during fermentation in comparison with continuous dark or blue illumination. b) SEM images show that pulse illumination improves the development of surface biofilm on the fermentation carriers. Scale bars are 20 µm.

To investigate this, we quantified the biofilm formation process during immobilized fermentation experiments and assessed the yield of *L*‐threonine produced by cocultures of nMagHigh and pMagHigh expressing strains (*E. coli* W1688 ΔycgF) under different illumination conditions, including dark, blue light, and pulsed illumination (5:20). The result of crystal violet staining assay revealed that under pulse conditions there was a significant (27.4%) increase in biofilm mass after 24 h compared to the dark treatment (Figure S8a,b, Supporting Information). Analogously, we produced under different illumination the biofilms on a cotton fiber carrier in a batch biofilm bioreactor, which is suitable for industrial scale‐up, and measured *L*‐threonine production and glucose consumption (**Figure** **7**a). Under constant and pulsed illumination, we observed that from batch to batch the *L*‐threonine yield increased and the fermentation period stabilized gradually after three cycles, both indicative of a more mature biofilm forming. In the seventh batch of immobilized fermentation in the dark, *L*‐threonine yield was 11.47 g L^−1^ with a fermentation period of 34 h marked by low glucose levels. Under blue light conditions in the same batch, the *L*‐threonine yield increased by 16.22% to 13.33 g/L and the fermentation period decreased by 3 to 31 h compared to the dark condition. The highest *L*‐threonine yield of 15.61 g L^−1^ was achieved under pulse illumination, resulting in a 36.09% increase compared to dark and a fermentation period reduced by 6 hours, totaling 28 h. To further validate the effectiveness of this strategy, we examined the biofilm structure on the carriers at different time points using scanning electron microscopy (SEM) (Figure 7b). The SEM images after the first, second, third, fourth, and seventh batches of immobilized fermentation, qualitatively show a gradual increase in the total amount of biofilms from batch to batch, where pulsed illumination produced a greater mass of biofilms which formed within a shorter time. In contrast, there was no effect of light illumination on the *L*‐threonine yield nor on the fermentation period in cultures of free‐floating bacteria (Figure S8c, Supporting Information). Overall, in the immobilized fermentation system, both *L*‐threonine yield was higher and the fermentation periods were shorter under pulsed light illumination than in the dark or under constant illumination. Therefore, this method holds great potential for enhancing fermentation efficiency and reducing fermentation costs for industrial fermentation processes.

## 3 Conclusion

In this study, we tuned bacterial cell‐cell adhesion dynamics to alter bacterial aggregation and behavior using photoswitchable adhesions. We showed that pulsed light leads to higher aggregation levels along with bigger and more compact clusters of bacteria compared to continuous light illumination. Using individual‐based simulations, we could understand this being the result of different bacteria cell‐cell adhesion dynamics, which depend on the interplay of the decay rate of the adhesins with the off‐time of the pulsed illumination. Thus, analogous to different states of matter, bacterial aggregates display gas‐like, liquid‐like, and solid‐like behavior in the dark, under pulsed illumination, and under continuous illumination, respectively. We subsequently demonstrate that bacterial aggregates with liquid‐like behavior have higher particle intermixing and activate quorum sensing earlier. Moreover, biofilms grew thicker and denser under pulse illumination than under constant blue light or in the dark, highlighting the importance of cell‐cell adhesion dynamics in biofilm maturation. We then applied this knowledge to cocultures of different pairs of auxotrophic bacteria, where biofilm growth was significantly affected by light conditions. Here, some pairs indeed did best under pulsed light, whereas others grew best under dark or constant light conditions. Hereby showing that, depending on the system, different light conditions can be employed to optimize cocultures. Finally, we improved *L*‐threonine production of biofilms in bulk bioreactors by subjecting the biofilms to pulse‐illumination, leading to better biofilms, and subsequently higher *L*‐threonine yield and shorter fermentation times. Hereby, showing that pulse‐illumination can be effectively applied in biotechnological and industrial settings to optimize biofilm formation.

The parallels between phase transitions, colloidal self‐assembly, and the assembly of multicellular structures are remarkable. Like for colloidal particles, it is possible for bacterial aggregates to transition from solid‐like DLCA under kinetic control to liquid‐like RLCA under thermodynamic control by simply increasing the exchange rate between the colloids/bacteria. Moreover, we found similar behavior also during the self‐assembly and self‐sorting with photoswitchable cell‐cell adhesions in mammalian cells.^[^ ^46^ ^]^ Yet, it should be noted that at later stages of biofilm development, the analogy is distorted by the growth of bacteria, the production of extracellular polymers, and cell differentiation within the biofilm over time. From this perspective, the maturation of a biofilm could be viewed as a transition from gas‐like planktonic bacteria to liquid‐like microcolonies that become more solid with time. Differently from the light‐induced adhesion used here, bacteria alter these cell‐cell interaction dynamics through the expression of surface proteins and secreted extracellular polymers.^[^ ^8^ ^]^ Nonetheless, also within the mature biofilm, the cell‐cell adhesion dynamics are clearly of significance, as they determine bacterial mobility and colony fluidity and the dispersion for future colonization.

Optogenetics is increasing in its importance to control bioproduction, cocultures of bacteria, and structuring biofilms, as light is a cheap stimulus that provides superb spatiotemporal and quantitative control.^[^ ^47^, ^48^ ^]^ Illumination with external periodic light stimuli is straightforward to implement in existing reactor designs with no concerns about contamination or off‐targeting. Existing tools already allow for optogenetic modulation of bacterial growth to maintain stable co‐cultures over long periods^[^ ^49^ ^]^ and specifically tune the activation of different pathways as desired.^[^ ^44^, ^50^, ^51^ ^]^ Moreover, the light‐inducible production of various biofilm‐forming molecules also makes it possible to pattern biofilms with high fidelity.^[^ ^27^, ^28^, ^29^, ^30^, ^31^, ^52^ ^]^ The here presented tuning over cell‐cell adhesion dynamics adds another modality to enhance biofilm formation and cocultures that is regulated directly at the level of adhesions and not gene expression. This allows for faster on/off dynamics that are required at the time scale of microcolony formation and biofilm compacting and leaves the possibility to combine with other approaches to modulating biofilms.

In summary, the dynamics of cell‐cell adhesion are just as important as the interaction strength in determining the bacterial behavior including quorum sensing and biofilm formation. The photoswitchable bacterial cell‐cell adhesions provide a straightforward approach to altering the cell‐cell adhesion dynamics and the associated processes systematically. Here, we demonstrate how important cell‐cell adhesion dynamics are in engineering bacterial communities at the micro‐ and mesoscopic scale with potential use in research and industrial applications.

## 4 Experimental Section

### Plasmids, Bacteria and Materials

The plasmids pB33‐nMagHigh‐eCPX or pB33‐pMagHigh‐eCPX (chloramphenicol resistant, *L*‐arabinose inducible) were previously described.^[^ ^33^ ^]^ Plasmids expressing GFP, mCherry, eCFP, and eYFP in a pTrc99A vector (ampicillin resistant, IPTG inducible) and the quorum sensing plasmid pUA66‐P*lsr*‐eGFP^[^ ^8^ ^]^ (kanamycin resistant) were a gift from Prof. Victor Sourjik (Max Planck Institute for Terrestrial Microbiology). *E. coli* K‐12 MG1655 was purchased from DSMZ and the auxotrophic BL21 (DE3) *E. coli* strains RF2 (Addgene plasmid # 62070, T), RF6 (Addgene plasmid # 62074, P), RF10 (Addgene plasmid # 62076, K), RF11 (Addgene plasmid # 61961, M), and RF12 (Addgene plasmid # 62077, W) were a gift from Robert Gennis and Toshio Iwasaki.^[^ ^53^ ^]^

All chemicals were purchased from Sigma‐Aldrich. Eight‐well slides (µ‐Slide, 8‐well glass bottom) are from ibidi and 96‐well microplates are from Greiner Bio‐One. The illumination setup used was built with off‐the‐shelf components including 460 nm LED strips connected to a power regulator, which controlled the intensity, and a programmable timer plug which regulated the pulsing frequency.

### Bacterial Strains

*E. coli* K‐12 MG1655 was co‐transformed with plasmids expressing one of the photoswitchable adhesins (pB33‐nMagHigh‐eCPX or pB33‐pMagHigh‐eCPX) and one of the plasmids expressing a fluorescent protein (pTrc99A‐ mNeonGreen, mCherry, eYFP or eCFP) or the quorum sensing reporter plasmid (pUA66‐P*lsr*‐eGFP). All overnight cultures were grown at 37 °C and 250 rpm covering the tubes in aluminum foil to keep samples dark.

### Aggregation Assay and Analysis

A single colony of the desired strains (nMagHigh‐eCPX/pTrc99A‐mNeonGreen or pMagHigh‐eCPX/ pTrc99A‐mCherry) were picked and cultured in LB medium (10 ml) in the presence of the appropriate antibiotics and inducers (50 µg ml^−1^ ampicillin, 35 µg/ml chloramphenicol, 0.04% *L*‐arabinose, 100 µM IPTG) overnight. Next, the bacteria were diluted in phosphate saline buffer (PBS) to an OD_600_ = 0.12, and cultures expressing complementary photoswitchable adhesins were mixed in a 1:1 ratio. Aliquots of 300 µL were added into 8‐well slides and incubated at 22 °C under the desired illumination setting (460 nm LED, 300 µW cm^−2^) for 2 h without shaking. All experiments were performed in 3 biological replicates with 3 technical replicates each.

Microscopy images were acquired on an inverted confocal laser scanning microscope (CLSM, Leica TCS SP8) equipped with a 488 and 552 nm laser for imaging the mNeonGreen and mCherry respectively, and a 40x water‐immersion objective. For each sample, 25 images (290 × 290 µm each, total area ca. 2.1 mm^2^) were acquired in the mNeonGreen and mCherry channels. All images were processed in FIJI.

To analyze bacterial aggregation, the images in the GFP and mCherry channels were merged and converted into a binary image. All bacteria (area ≥ 2 µm^2^, area of a single bacteria) and clustered bacteria (area ≥ 35 µm^2^, area of at least 15 bacteria) were detected using the Analyze Particles function in FIJI. The aggregation ratio is equal to the total area occupied by clustered divided by the total area occupied by all bacteria. The fractal dimension of the bacterial clusters (area ≥ 35 µm^2^) was quantified using the FracLac plugin for FIJI.^[^ ^54^, ^55^ ^]^

### Individual‐Based Simulations

We model our bacteria as spherocylindrical particles as previously implemented^[^ ^56^ ^]^ with diameter *D* = 1 µm and a length taken from a uniform distribution between 1 and 2 µm. Because the bacteria in the experiments tend to sediment to the bottom of the wells quite quickly and only the bottom layer is imaged, we initialize the particles in a thin slab right above the surface with periodic boundary conditions on the x and y axis (2000 particles in a 110 × 110 × 5 box). For each timestep, the forces on each particle are calculated, and their positions are updated accordingly, using an overdamped dynamics model. The amount of timesteps per experimental minute is set to 25 000.

The particles are subject to Brownian motion which is implemented by giving each end of the particles a kick in a random direction and with a magnitude taken from a normal distribution with mean 0 and standard deviation *σ*_bf_. A suitable value for *σ*_bf_ was chosen by first, estimating the diffusion coefficient for a spherical particle of the same diameter, suspended in PBS buffer. Correcting for the fact that the diffusion takes place near a wall,^[^ ^57^ ^]^ and that the particles are spherocylindrical with an aspect ratio of three,^[^ ^58^ ^]^ gave us a diffusion constant of *D*_particle_ = 0.024 µm^2^ s^−1^. Measuring the MSD after one hour for 2000 particles we find that *σ*_bf _= 10^−3^ gives us an MSD of the expected ≈500 µm^2^ (Figure S3, Supporting Information). For each particle pair that is within reach of each other, the shortest distance between the two particles is determined. The magnitude of the adhesive force they experience depends on this distance *r* in the following way:

(2) $$F_{\mathrm{particle}}\;\left(r\right)=\;P\;\cdot \frac{r}{\;\alpha }\cdot \;e^{-\frac{\left(r+\alpha \right)}{\alpha }},\;r\;\geq 0$$

(3) $$F_{\mathrm{particle}}\;\;\left(r\right)=K\;\;\cdot \;r$$

where *α* determines the interaction range and *P* determines the amplitude of the attractive forces. If there is overlap between the particles, the force is taken to be in the form of a spring force with spring constant *K*. The resulting force is then distributed over the two end‐points of the particle inversely related to where the closest point is situated along the length of the particle. Because the membrane proteins are not more than a couple of nanometers across, we argue that the range of the interaction should be around twice their size (≈5 nm) The interaction strength should be quite strong, so that once the particles adhere to each other or to the surface, they would not come apart due to Brownian forces. Particles also interact with the surface; this interaction does not depend on the light conditions. The surface adhesion works on both ends of the particles separately because we know that surface interaction is mostly modulated on the ends of *E. coli*.^[^ ^59^ ^]^ The magnitude of the surface adhesion for each endpoint is calculated as follows:

(4) $$F_{\mathrm{surface}}\;\left(r\right)=\;S\cdot \;\frac{r}{\beta }\cdot \;e^{-\frac{\left(r+\;\beta \right)}{\beta }},\;r\geq 0$$

(5) $$F_{\mathrm{surface}}\;\left(r\right)=\;K\;\cdot \;r,\;r<0$$

we chose the magnitude of this adhesion such that it is weaker than the adhesion between particles but stronger than the Brownian forces because we know from the experiments that clusters can pull single particles off the surface. By comparing the aggregation curves under constant blue light over a span of 6 hours between the experiments^[^ ^33^ ^]^ and simulations we settled on values *P* = 5–10^−3^ and *S *= 5–10^−4^ with ranges *α* = 5 nm and *β* = 10 nm (FigureS4a,b, Supporting Information). Please note that, even though higher adhesive strengths gave a better fit to the data, this resulted in artifacts in the simulations in the form of fast‐spinning particles (not shown). Additionally, we show that changing the range α does not affect the results significantly (Figure S4c, Supporting Information).

Four times every experimental minute, all the particle coordinates and orientations were logged. These were then used to create images of the bottom 1.2 µm slab of the simulation box, which is the *z* resolution of the microscope used, using Python. The images were then analyzed using ImageJ in the same way that is used for the experimental images (Analyze Particles, etc.). We use this data to track the aggregation ratio and cluster size over the course of the simulation experiments. We also measure the fractal dimension using Fraclac. We also tracked which particles were within each particle's vicinity by logging the neighbors' particle ID's.

### Quorum Sensing Activation

Single colonies of the desired strains (nMagHigh‐eCPX/pUA66‐P*lsr*‐eGFP or pMagHigh‐eCPX/pUA66‐P*lsr*‐eGFP) were picked and cultured in LB broth (10 mL) with the respective antibiotics (50 µg mL^−1^ Ampicillin, 35 µg mL^−1^ chloramphenicol) incubated. The overnight cultures were diluted 1:1000 into 10 mL LB medium supplemented with antibiotics and 0.04% *L*‐arabinose. The bacteria were cultured in the dark at 37 °C, at 200 rpm for 3–4 h until OD_600_ = 0.6 was reached. Both cultures were diluted with PBS to OD_600_ = 0.12 and mixed in a 1:1 ratio. Aliquots of 300 µl were added into an 8‐well slide and incubated at 22 °C under the respective illumination setting for 2 h. Confocal microscopy images were acquired in the GFP channel and the eGFP reporter signal was quantified with FIJI using the Integrated Density measurement. All experiments were performed in 3 biological replicates with 3 technical replicates each.

### Biofilm Formation

Overnight cultures of the desired strains (nMagHigh‐eCPX/pTrc99A‐eCFP or pMagHigh‐eCPX/ pTrc99A‐eYFP) were grown in the presence of antibiotics and inducers (50 µg mL^−1^ Ampicillin, 35 µg mL^−1^ Chloramphenicol and inducers 0.04% *L*‐arabinose, 100 µM IPTG). The next day, the cultures were diluted into LB medium supplemented with antibiotics and inducers to reach an OD_600_ = 0.01. The two strains were mixed in a 1:1 ratio, and 300 µl aliquots were transferred to 8‐well slides. The samples were incubated at 37 °C without shaking under the respective illumination setting for 48 h. The samples were rinsed three times with water, and z‐stacks were acquired using the CLSM using the 405 and 488 nm lasers for fluorescence activation of eCFP (475–495 nm) and eYFP (540‐560 nm), respectively. The biofilms were 3D reconstructed using the 3D Viewer from the Leica LAS X software. Details of the biofilm morphology were analyzed using the COMSTAT 2 plug‐in^[^ ^60^ ^]^ in FIJI. All experiments were performed in 5 biological replicates.

For crystal violet staining we used a modified version of previously described protocols,^[^ ^61^ ^]^ where the experiment was repeated as described above but the bacteria were only transformed with nMagHigh‐eCPX or pMagHigh‐eCPX and 200 µL aliquots of the coculture were added into 96‐well round bottom plates. After 48 h incubation, the samples were gently rinsed three times with water, each well then received 200 µL of a 0.1% solution of crystal violet in water and were incubated for 15 min at room temperature. Then, the wells were rinsed three times with water and the remaining crystal violet was solubilized by adding 200 µL of 30% acetic acid in water. Finally, the solution was diluted 1:5 with water and the absorbance at 550 nm was measured in flat‐bottomed transparent 96‐well microplates using a plate reader (TECAN Spark). The experiments were conducted in three biological replicates.

### Auxotrophic Bacterial Cocultures

Overnight cultures of the desired auxotroph strains (RF2, RF6, RF10, RF11, RF12 were a gift from Robert Gennis and Toshio Iwasaki; Addgene #: 62070, 62074, 62076, 61961, and 62077, respectively) transformed with the photoswitchable adhesins and a fluorescent label (nMagHigh‐eCPX/pTrc99A‐eCFP or pMagHigh‐eCPX/ pTrc99A‐eYFP) were grown in the presence of antibiotics and inducers (50 µg mL^−1^ Ampicillin, 35 µg mL^−1^ Chloramphenicol and inducers 0.04% *L*‐arabinose, 100 µM IPTG) were spun and resuspended in minimal M9 media with the same antibiotics and inducers, to reach OD_600 _= 0.01 (≈10^7^ cells mL^−1^). Combinations of these two strains were mixed in a 1:1 ratio and 500 µL aliquots were transferred to flat‐bottomed transparent 48‐well microplates and incubated at 37 °C and 200 rpm orbital shaking, under the respective illumination setting for 48 h, when the final OD_600_ was measured and the growth fold was calculated respective to the initial bacterial concentration. All experiments were performed in 3 biological replicates.

Biofilm growth of auxotrophic strains was similar as described above, but 300 µL aliquots of selected cocultures were placed on 8‐well slides and incubated at 37 °C without shaking under the respective illumination setting for 48 h. Excess media was pipetted out of the wells without disturbing the delicate biofilms and z‐stacks were acquired using the CLSM, which were analyzed as described above.

### Free‐Cell Fermentation, Immobilized Fermentation, and Carrier SEM Analysis

The study involved testing cocultured *E. coli* strains, specifically the ΔycgF + nMagHigh and ΔycgF + pMagHigh, through free‐cell fermentation and immobilized continuous fermentation. Cotton fiber carriers previously characterized for industrial fermentation,^[^ ^62^, ^63^ ^]^ were taken from the fermentation batches and observed through SEM electron microscopy. The methods and instrumentation were employed as previously described,^[^ ^45^ ^]^ illumination conditions set to dark, blue (450 nm; 500 lux), and 5:20 ON:OFF pulses.

Co‐cultured *E. coli* ΔycgF + nMagHigh and ΔycgF + pMagHigh were subjected to free‐cell fermentation, immobilized continuous fermentation, and their carriers were photographed by SEM electron microscopy. The methods and instrumentation used in the above experiments were also modified from previous literature^[^ ^45^ ^]^ to include our current illumination settings.

## Author Contributions

J.J.Q.H., F.C., and S.V.W. designed the experiments; J.J.Q.H. performed the experiments with support from F.C., analyzed the data, and prepared the figures. R.L. conducted the computational simulations under the guidance of T.I.. S.S., W.S., and Y.C. characterized *L‐threonine* production under different conditions. All authors read and reviewed the results and approved the final version of the manuscript.

## Conflict of Interest

The authors declare no conflict of interest.

## Supporting information

Supporting Information

Supporting Information

Supporting Information

Supporting Information

## Data Availability

The data that support the findings of this study are available from the corresponding author upon reasonable request.
