# Supplementary material for: Optogenetic Control of Bacterial Cell‐Cell Adhesion Dynamics: Unraveling the Influence on Biofilm Architecture and Functionality
Source: Adv Sci (Weinh). 2024 Apr 13;11(23):2310079. doi: 10.1002/advs.202310079 (PMC11187914; doi:10.1002/advs.202310079)
Supplement: Supplementary file 1 — Supporting Information [file ADVS-11-2310079-s001.pdf]

ADVANCED SCIENCE
Open Access

Supporting Information

for *Adv. Sci.*, DOI 10.1002/advs.202310079

Optogenetic Control of Bacterial Cell-Cell Adhesion Dynamics: Unraveling the Influence on Biofilm Architecture and Functionality

*Juan José Quispe Haro, Fei Chen, Rachel Los, Shuqi Shi, Wenjun Sun, Yong Chen, Timon Idema and Seraphine V. Wegner**

Supporting Information

Optogenetic Control of Bacterial Cell-Cell Adhesion Dynamics: Unraveling the Influence on Biofilm Architecture and Functionality

Juan José Quispe Haro,[1] Fei Chen,[1-2] Rachel Los,[3] Shuqi Shi,[4,5] Wenjun Sun,[4,5] Yong Chen,[4,5] Timon Idema,[3] Seraphine V. Wegner[1]*

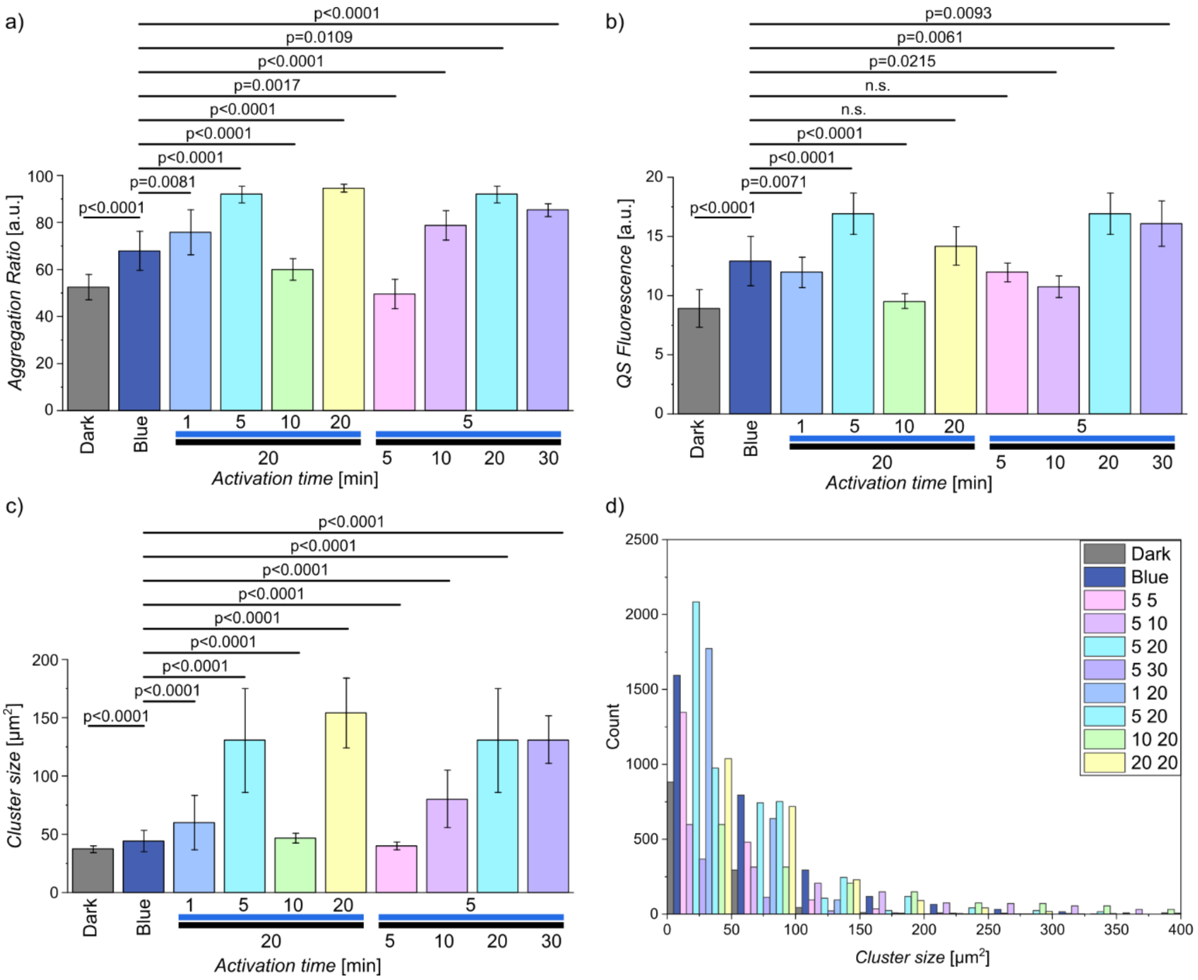

Figure S1. (a) Average aggregation ratio. (b) Average GFP fluorescence of QS reporter bacteria. (c-d) Cluster size distribution and mean. p-Values correspond to individual t-tests.

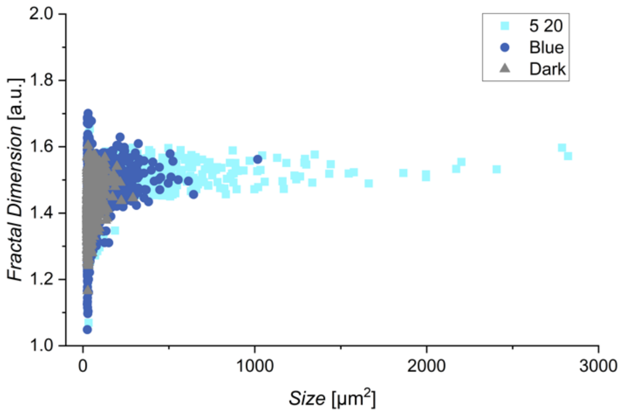

Figure S2. Correlation of cluster size with fractal dimension.

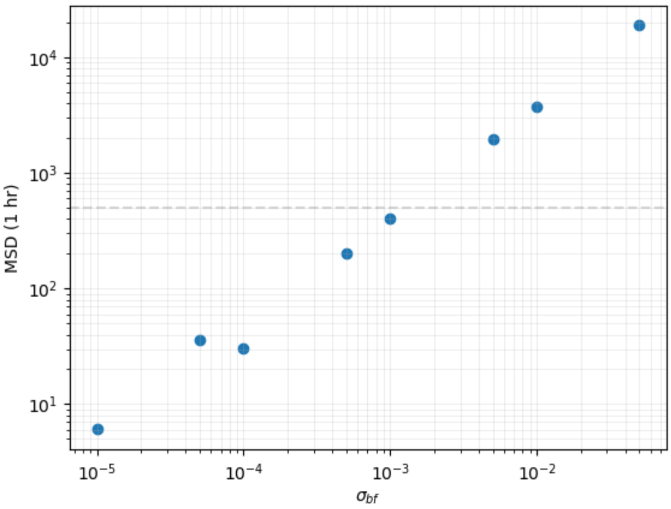

Figure S3. Mean-squared displacement (MSD) of particles after 1 hour of darkness for different values of σ_{bf}. Dashed line represents the expected MSD for the bacteria at 500 μm^2.

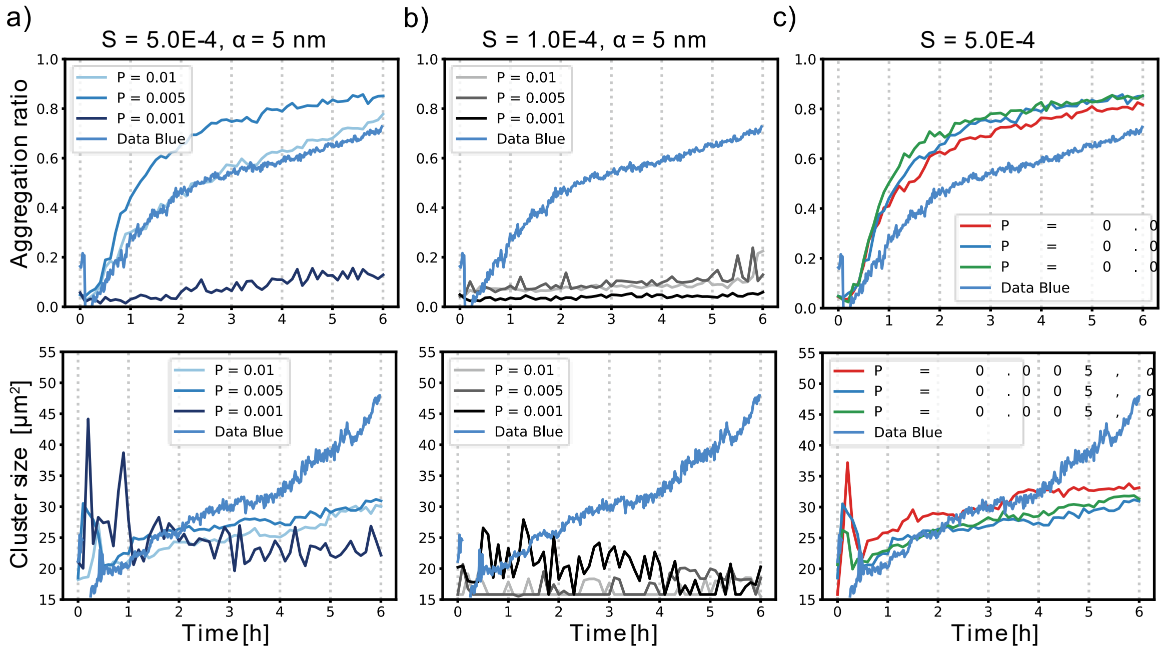

Figure S4. Determining the parameters of the interaction parameters. Aggregation ratio and cluster size curves for different values of *P*, *S* and *α*.

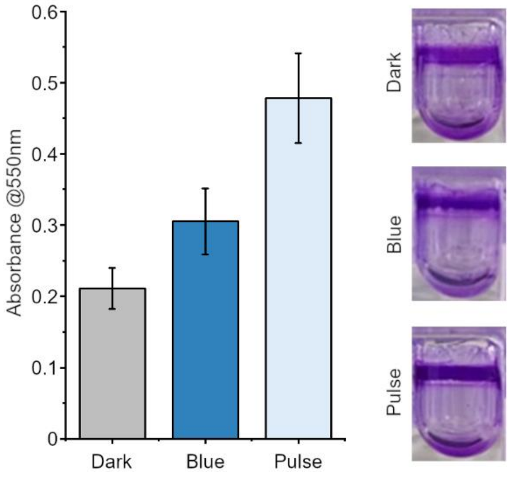

Figure S5. Pulse illumination enhances biofilm formation of *MG1655 E. coli K-12* expressing nMagHigh and pMagHigh adhesins. Biofilm grown in 96-well plates were stained with crystal violet to quantify the total biofilm biomass by light absorption spectroscopy at 550 nm. Experiments were performed in biological triplicates.

	Dark	Blue	Pulse
Biomass (μm^3 μm^{-2})	12.459	15.802	60.448
Maximum diffusion distance (μm):	13.769	13.918	27.431
Average diffusion distance (μm):	0.608	0.768	5.244
Average fractal dimension:	1.145	1.067	1.168
Average thickness (μm):	16.000	31.000	43.000
Roughness Coefficient (Ra*):	0.928	0.465	0.096
Surface Area (μm^2):	5.9×10^5	1.1×10^6	1.7×10^6
Surface to biovolume ratio (μm^2 μm^{-3}):	0.831	1.305	0.344

Figure S6. Biofilm analysis from *MG1655 E. coli K-12* expressing nMagHigh or pMagHigh. Pulse illumination enhances biofilm formation and has a significant effect on all the properties analyzed using COMSTAT.

Kn-Mp Coculture	Dark	Blue	Pulse
Biomass (μm^3/μm²)	54.227	38.852	32.851
Maximum diffusion distance (μm):	18.997	13.234	12.978
Average diffusion distance (μm):	1.755	1.607	1.125
Average fractal dimension:	1.127	1.150	1.157
Maximum thickness (μm):	103.500	68.000	66.667
Surface Area (μm²):	1.2E+07	9.1E+06	7.3E+06
Surface to biovolume ratio (μm²/μm^3):	2.589	2.773	3.454
Average volume of colonies at substratum (μm^3):	4.1E+06	3.3E+06	2.7E+06

Wn-Kp Coculture	Dark	Blue	Pulse
Biomass (μm^3/μm²)	33.663	36.437	30.799
Maximum diffusion distance (μm):	13.940	13.594	12.996
Average diffusion distance (μm):	1.412	1.266	1.261
Average fractal dimension:	1.163	1.160	1.179
Maximum thickness (μm):	61.667	69.000	60.333
Surface Area (μm²):	7.4E+06	8.9E+06	6.6E+06
Surface to biovolume ratio (μm²/μm^3):	2.593	2.906	2.537
Average volume of colonies at substratum (μm^3):	2.7E+06	3.0E+06	2.5E+06

Pn-Kp Coculture	Dark	Blue	Pulse
Biomass (μm^3/μm²)	37.980	28.673	57.085
Maximum diffusion distance (μm):	15.329	14.428	14.931
Average diffusion distance (μm):	2.016	1.893	1.391
Average fractal dimension:	1.184	1.173	1.199
Maximum thickness (μm):	63.000	59.333	118.000
Surface Area (μm²):	7.1E+06	7.2E+06	1.5E+07
Surface to biovolume ratio (μm²/μm^3):	2.233	2.379	3.009
Average volume of colonies at substratum (μm^3):	2.8E+06	2.9E+06	4.6E+06

Pn-Mp Coculture	Dark	Blue	Pulse
Biomass (μm^3/μm²)	34.390	53.082	72.680
Maximum diffusion distance (μm):	11.793	14.484	17.742
Average diffusion distance (μm):	1.287	1.311	1.964
Average fractal dimension:	1.149	1.163	1.157
Maximum thickness (μm):	68.333	107.667	128.000
Surface Area (μm²):	8.4E+06	1.3E+07	1.8E+07
Surface to biovolume ratio (μm²/μm^3):	2.895	2.813	2.865
Average volume of colonies at substratum (μm^3):	2.8E+06	4.4E+06	6.1E+06

Figure S7. Biofilm analysis from *E. coli* auxotroph strains expressing nMagHigh or pMagHigh. Pulse illumination allows for tunable responses in biofilm growth and properties of consortia cocultures.

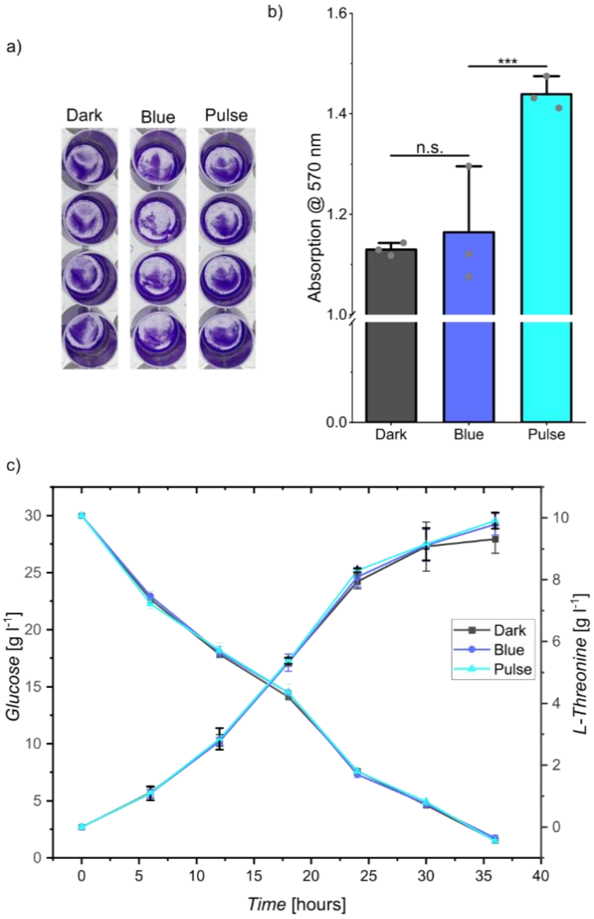

Figure S8. Effects of various light frequencies on biofilm formation in *E. coli*. (a) *E. coli* ΔycgF + nMagHigh or pMagHigh strains were co-inoculated into a 24-well plate, incubated at 37°C in dark, blue and 5: 20 pulse conditions for 30 h and then imaged after crystal violet staining before washing, and the corresponding absorbance at 570 nm (b) (Student's t-test, n.s.: not significant; ***: $p < 0.001$). Experiments were performed in biological triplicates. (c) Effect of three light frequencies on *L*-threonine production and glucose consumption in free-cell fermentation of *E. coli* ΔycgF expressing the surface adhesins.
